# Persian version of the work design questionnaire: measurement of its psychometric characteristics

**DOI:** 10.1186/s40359-022-00922-5

**Published:** 2022-09-19

**Authors:** Zeinab Rasouli Kahaki, Vahid Gharibi, Mojtaba Keshavarz, Rosanna Cousins, Hamidreza Mokarami

**Affiliations:** 1 Student Research Committee, Department of Ergonomics, School of Health, Shiraz University of Medical Sciences, Shiraz, Iran; 2 School of Public Health, Shahroud University of Medical Sciences, Shahroud, Iran; 3 Department of Psychology, Liverpool Hope University, Liverpool, UK; 4 Department of Ergonomics, School of Health, Shiraz University of Medical Sciences, Shiraz, Iran

**Keywords:** Work design, Job characteristics, Work characteristics, Validity, Reliability, Iranian employees

## Abstract

**Background:**

Work design questionnaire (WDQ), as a comprehensive and integrative tool, is one of the most important instruments frequently used to assess work characteristics. The aim of this study was to measure the psychometric characteristics of the Persian version of WDQ.

**Methods:**

Translation and cross-cultural adaptation procedures were applied in translating the original WDQ into Persian. A total of 270 participants participated in this study. The validity of the questionnaire were measured using face validity, content validity, convergent validity, and construct validity based on confirmatory factor analysis (CFA). Reliability was assessed through internal consistency.

**Results:**

Mean content validity index was 0.95. The CFA results indicated support for a 21-factor solution. There were significant correlations between dimensions of WDQ and both job satisfaction and perceived stress. Cronbach's alpha of all items was 0.87.

**Conclusion:**

Results indicated that the WDQ exhibited very good psychometric properties and can be applied as a useful tool to assess work characteristics among Iranian employees. Accordingly, the authors recommend its administration in future studies. The work characteristics was significantly associated with job satisfaction and job stress. Therefore, improved work design would reduce negative consequences, such as job stress, and increase positive behaviors, such as job satisfaction.

## Background

Work design is a major controversial topic in organizational science studies. Besides describing how to structure, approve, and revise the work requirements, tasks, and roles, it explains their impact on individuals, groups, and organizational outcomes [1]. Work design affects behavioral outcomes, such as performance, efficiency, and work-related absence, psychological outcomes (such as job satisfaction, motivation, stress, and burnout), and physical consequences (such as hypertension, cardiovascular diseases, and even death) [2–6].

Smith [7] and Babbage [8] first introduced the principles of work design by describing the concept of division of labor and its effect on productivity and efficiency [9]. Herzberg’s 2-factor theory (1959) was another step that played a significant role in work design [10]. According to this theory, two sets of factors (hygiene and motivation) affect performance. The idea is that the absence of hygiene factors will result in a lack of job satisfaction. On the other hand, motivation factors provide job satisfaction and motivation. Both factors are important in supporting best work design. Several other theories on work design have also been introduced in the past four decades, indicating a wide spectrum of personal, group, and organizational outcomes that they can affect [11, 12]. Nevertheless, despite extensive research investigating the importance of work design, relatively few studies have evaluated job characteristics and factors that contribute to workplace design.

Self-report questionnaires are among the most important and widely applied tools in work design. One of the most applied tools is the Job Diagnostic Survey (JDS). Developed in 1975, this contains five dimensions of motivational job characteristics, but ignoring multiple job characteristics is one of the major pitfalls of the JDS [12, 13]. To address this, Campion [14] introduced the Multimethod Job Design Questionnaire (MJDQ) [14]. Although this tool covers a broader range of job characteristics, the MJDQ has structural problems [14]. For instance, Edward et al. [15] found that a 10-factor structure is more appropriate than the 4-factor structure proposed by Campion. They also noted some conceptual problems regarding its keywords [15].

Ignoring technological developments and modern aspects of work is a common problem of work design questionnaires [12]. In response to previous problems, Morgeson and Humphrey [9] proposed the Work Design Questionnaire (WDQ), which is a comprehensive, integrative tool. According to its authors, the WDQ is needed for 3 reasons: (1) it consists of both task-oriented and attribute-oriented measures, (2) it covers a comprehensive and balanced set of job characteristics, and (3) it is able to integrate different perspectives. The WDQ contains 77 items categorized into three dimensions of work: motivational, social, and context. These dimensions cover 20 different fields, such as independence at work, importance and identity of duty, complexity, job skills, physical needs, and ergonomics [16]. As a powerful tool to measure work design, WDQ is compatible with new concepts and covers a wide range of variables related to job characteristics.

The WDQ has been successfully translated into several different languages, such as German, Chinese, Polish, and Spanish [17–20]. To the best of our knowledge, WDQ has not been formally translated into Persian. Hence, its psychometric properties have not yet been evaluated in Iran. Accordingly, this study aimed to assess the psychometric characteristics of our Persian version of WDQ. The aim of this study was to measure the psychometric characteristics of the Persian version of WDQ.

## Methods

### Design and study population

This cross-sectional study was conducted using employees working in an industrial estate during the summer and fall of 2021. All workers with at least 1 year of work experience were initially invited and a total of 286 out of the 462 workers (response rate: 61.9%) participated in the study. In the initial stage, 16 questionnaires were removed from the study due to missing data, misleading responses and authentication responses*.* Therefore, 270 questionnaires were statistically analyzed. The participants completed the survey anonymously after providing informed consent. Participants were aware of the purposes and hypotheses of the study. The study was conducted in 2021, according to the principles of the Declaration of Helsinki, and was approved by the Research Ethics Committee of Shiraz University of Medical Sciences (IR.SUMS.REC.1398.537).

The purpose of the study, how to answer the questions, and the ethical obligations of the researchers regarding the completed questionnaires was explained to the employees. Then, a questionnaire was distributed to each consenting participant with information on how to return it to the researchers after completing it. Participation in this research was entirely voluntary, the questionnaires were anonymous, and the results were analyzed in terms of the information of all respondents.

### Translation and cross-cultural adaptation

To respect the intellectual property rights of the instrument, first, the approval of the original authors was obtained for translation into Persian. Based on the process proposed by Beaton et al. [21] for translation and cross-cultural adaptation, forward translation was performed by two ergonomics experts fluent in the English language. The research team then compared the translated Persian versions, and differences and ambiguities were discussed. Afterward, translated items were reviewed in terms of semantics, cultural adaptation, and terminology. Eventually, a single Persian provisional version was developed. The next step was the backward translation of the Persian provisional version by two language experts fluent in English unaware of the original English version. The research team revised the translated versions, and a single English provisional version was obtained by combining them. Afterward, this version, along with ambiguities and inconsistencies, was sent to the developers of the WDQ for clarification and conditions of acceptability. The proposed revisions were made, and the final version was prepared for psychometric evaluations. The Persian version is available on the developer Prof. Morgeson’s questionnaire website (http://www.morgeson.com/wdq.html).

### Measuring validity and reliability

#### Face and content validity

Face validity is the extent to which a test can cover the concept it intends to measure [22]. In this study, to achieve face validity we sought the opinions of 10 university professors (ergonomics and industrial and organizational psychology). That is, the professors were asked to evaluate each of the items in terms of comprehensibility, wording, interpretations, cultural issues, and clarification. In addition, a sample of 15 employees from the participant pool was selected to assess the questionnaire in terms of potential ambiguities and understandability of items. After minor revisions, content validity was evaluated using the content validity index (CVI). Ten experts in ergonomics, occupational health, and industrial and organizational psychology were asked to comment on each item separately [22]. For CVI, a score higher than 0.79 is favorable, 0.7–0.79 indicates the need for revision, and less than 0.7 is unacceptable [23].

#### Construct validity

Construct validity of the questionnaire was assessed using confirmatory factor analysis (CFA) based the maximum likelihood estimation (MLE) method. The model fit was assessed using the root mean square error of approximation (RMSEA), the comparative fit index (CFI), the incremental fit index (IFI) and the chi-square/degrees of freedom ratio (χ^2^/*df*). If the RMSEA is less than 0.08, CFI value is 0.9 or higher, the IFI value is 0.8 or 0.9, and the χ^2^/*df* is less than 3, then the fit of the model is appropriate [24].

Based on previous studies, five models of 4, 18, 19, 20, and 21 factors were examined in this study. The 4-factor model contains 4 wide categories of work characteristics, including those related to task, knowledge, social, and work context. The 18-factor model covers the work characteristics without any divisions, including autonomy and interdependence, each as a unique factor. The 19-factor model divides interdependence into 2 components of received and initiated. The 20-factor model contains 3 elements of autonomy, including work scheduling, decision making, and work methods. The 21-factor model also includes identified components containing interdependence and autonomy.

#### Incremental validity

Incremental validity of the WDQ was assessed using Perceived Stress Scale (PSS-10) and Michigan Organizational Assessment Questionnaire Job Satisfaction Subscale (MOAQ-JSS). The PSS measure the degree to which situations are appraised as stressful during the past month [25]. It uses a 5-point Likert scale, ranging from 0 (never) to 4 (always). Three versions of PSS are available that comprise 4, 10, and 14 items. Most prior studies have used the 10-item version, as it has fewer items than the original 14-item version, with similar psychological properties [26, 27]. The MOAQ-JSS measures job satisfaction using three items using a 7-point Likert scale [28].

#### Convergent/discriminant validity

The average variance extracted (AVE) and the maximum shared squared variance (MSV) were used to assess convergent validity and discriminant validity, respectively. The AVE is “the average variance extracted is calculated as the mean variance extracted for the item loading on a [factor] and is a summary indicator of convergence” and values ≥ 0.5 confirms the convergent validity [29]. On the other hand, discriminant validity is confirmed when the AVE is greater than the MSV [29].

#### Reliability

Cronbach’s alpha coefficient and composite reliability (CR) were used to assess the internal consistency of the WDQ. A Cronbach’s alpha and CR value of 0.70 or higher is considered acceptable [30]. In addition, the item-to-total correlation and Cronbach’s alpha if item deleted were also calculated separately. For each item, the item-to-total correlation should be higher than 0.3 to be considered acceptable [31].

### Statistical analysis

Data analysis was conducted using SPSS version 23 and AMOS version 23 (SPSS Inc, Chicago, Ill, USA). The Kolmogorov–Smirnov test was applied to test for normal distribution. Also, Mardia’s coefficient (based on critical ratio) was used to determine multivariate normality. Critical ratio values were significantly < 5, which indicated that the data met assumptions of normal distribution [32]. A *P* value < 0.05 was considered statistically significant.

## Results

Participants’ mean age and work experiences were 35.18 ± 7.5 years (ranging from 21 to 61 years) and 10.38 ± 8.5 years (ranging from 1 to 35 years), respectively. Also, 83.5% were married. In addition, 52% were educated up to diploma, 35.5% up to bachelor’s degree, and 9.5% up to master’s degree or higher.

The mean and SD of various dimensions of WDQ are presented in Table 1. There were no floor and ceiling effects for any dimension. The CVI score of the whole scale was 0.95. Based on this score, content validity was excellent from the experts’ viewpoint.

**Table 1:** Means, standard deviations, and reliability of the WDQ (n = 270)

Dimensions	Item	Mean (SD)		Internal consistency	Average variance extracted	Maximum shared squared variance	Construct/composite reliability	Corrected item-total correlation	Cronbach's alpha if item deleted
		Item	Scale						
*Task characteristics*									
Work scheduling autonomy	W1	3.23 (1.29)	10.03 (3.14)	0.79	0.63	0.60	0.77	0.70	0.63
	W2	3.31 (1.27)						0.68	0.66
	W3	3.49 (1.19)						0.52	0.82
Decision-making autonomy	W4	3.24 (1.28)	9.33 (3.36)	0.86	0.61	0.66	0.70	0.73	0.81
	W5	3.10 (1.27)						0.78	0.76
	W6	3.0 (1.25)						0.70	0.83
Work methods autonomy	W7	3.34 (1.20)	9.83 (3.09)	0.77	0.59	0.69	0.81	0.65	0.64
	W8	3.10 (1.29)						0.57	0.73
	W9	3.39 (1.26)						0.59	0.71
Task variety	W10	3.46 (1.36)	14.40 (4.44)	0.90	0.70	0.28	0.84	0.76	0.87
	W11	3.67 (1.22)						0.81	0.86
	W12	3.66 (1.26)						0.81	0.86
	W13	3.62 (1.25)						0.72	0.89
Task Significance	W14	3.70 (1.20)	14.44 (3.94)	0.85	0.54	0.25	0.79	0.51	0.80
	W15	3.76 (1.16)						0.43	0.83
	W16	3.49 (1.18)						0.59	0.78
	W17	3.49 (1.21)						0.53	0.82
Task identity	W18	3.81 (1.06)	14.94 (3.68)	0.87	0.58	0.44	0.81	0.70	0.84
	W19	3.71 (1.15)						0.76	0.81
	W20	3.69 (1.09)						0.79	0.81
	W21	3.72 (1.04)						0.65	0.86
Feedback from job	W22	3.66 (1.06)	10.90 (2.50)	0.64	0.46	0.42	0.65	0.53	0.39
	W23	3.67 (1.05)						0.59	0.31
	W24	3.57 (1.18)						0.24	0.81
*Knowledge characteristics*									
Job complexity	W25	2.99 (1.27)	13.48 (3.68)	0.73	0.53	0.37	0.85	0.30	0.79
	W26	3.53 (1.23)						0.67	0.58
	W27	3.43 (1.23)						0.68	0.57
	W28	3.53 (1.24)						0.48	0.69
Information processing	W29	3.75 (1.10	14.55 (3.84)	0.82	0.61	0.50	0.61	0.57	0.80
	W30	3.67 (1.28)						0.71	0.74
	W31	3.84 (1.16)						0.70	0.74
	W32	3.30 (1.26)						0.60	0.79
Problem solving	W33	3.07 (1.25)	12.91 (3.67)	0.73	0.41	0.56	0.52	0.53	0.66
	W34	3.32 (1.2)						0.51	0.69
	W35	3.08 (1.3)						0.37	0.75
	W36	3.49 (1.20)						0.68	0.57
Skill variety	W37	3.64 (1.26)	14.34 (4.47)	0.93	0.70	0.47	0.82	0.81	0.91
	W38	3.57 (1.23)						0.87	0.89
	W39	3.53 (1.28)						0.84	0.90
	W40	3.61 (1.18)						0.80	0.92
Specialization	W41	3.44 (1.29)	14.12 (4.21)	0.88	0.52	0.40	0.69	0.76	0.84
	W42	3.47 (1.24)						0.77	0.83
	W43	3.65 (1.17)						0.76	0.84
	W44	3.56 (1.24)						0.67	0.87
*Social characteristics*									
Social support	W45	3.78 (1.05)	21.19 (4.47)	0.75	0.43	0.32	0.72	0.56	0.67
	W46	3.51 (1.24)						0.46	0.71
	W47	3.14 (1.27)						0.38	0.74
	W48	3.44 (1.14)						0.43	0.72
	W49	3.66 (1.0)						0.56	0.69
	W50	3.67 (1.03						0.53	0.69
Initiated interdependence	W51	3.65 (1.12)	21.52 (5.44)	0.82	0.57	0.55	0.77	0.66	0.77
	W52	3.70 (1.06)						0.72	0.71
	W53	3.63 (1.19)						0.65	0.78
Received interdependence	W54	3.60 (1.07)	10.55 (3.01)	0.84	0.56	0.48	0.83	0.71	0.76
	W55	3.58 (1.14)						0.77	0.70
	W56	3.37 (1.26)						0.63	0.85
Interaction outside organization	W57	2.80 (1.28)	12.39 (3.80)	0.79	0.71	0.46	0.81	0.75	0.67
	W58	2.95 (1.21)						0.69	0.70
	W59	3.03 (1.26)						0.69	0.70
	W60	3.61 (1.08)						0.32	0.86
Feedback from others	W61	3.69 (1.02)	10.78 (2.80)	0.81	0.61	0.29	0.84	0.70	0.70
	W62	3.59 (1.12)						0.70	0.69
	W63	3.51 (1.14)						0.58	0.82
*Work context*									
Ergonomics	W64	2.54 (1.27)	8.79 (2.80)	0.62	0.47	0.38	0.46	0.54	0.37
	W65	2.89 (1.24)						0.55	0.35
	W66	3.36 (1.20)						0.24	0.77
Physical demands	W67	3.45 (1.32)	10.60 (3.27)	0.83	0.78	0.23	0.82	0.79	0.66
	W68	3.49 (1.31)						0.75	0.71
	W69	3.66 (1.14)						0.55	0.89
Work conditions	W70	2.68 (1.45)	14.18 (4.74)	0.74	0.50	0.35	0.85	0.50	0.69
	W71	2.91 (1.30)						0.38	0.73
	W72	2.79 (1.33)						0.53	0.68
	W73	2.66 (1.34)						0.63	0.64
	W74	3.14 (1.37)	9.36 (3.31)					0.46	0.71
Equipment use	W75	3.33 (1.25)		0.83	0.58	0.41	0.84	0.73	0.71
	W76	3.19 (1.28)						0.73	0.71
	W77	2.84 (1.31)						0.59	0.85

### Construct validity

The fit indices of developed models are presented in Table 2. The 4-factor model presented a poor fit as its fitness indices were lower than the desired level. The 18-, 19-, and 20-factor models all showed adequate fits. The goodness-of-fit indices of χ^2^/df, RMSEA, and IFI were acceptable, and only CFI was lower than the desired level (i.e., 0.90). Finally, the 21-factor model was the best fit model, with all goodness-of-fit indicators acceptable.

**Table 2:** Result of confirmatory factor analyses (N = 270)

Model	χ^2^	Df	Ӽ^2^/df ratio	RMSEA	IFI	CFI
4-factor	6862	2796	2.45	0.07	0.72	0.72
18-factor	5189	2653	1.96	0.06	0.83	0.82
19-factor	4719	2600	1.82	0.05	0.89	0.87
20-factor	4821	2584	1.87	0.05	0.89	0.87
21-factor	4554	2571	1.77	0.04	0.91	0.91

*RMSEA* root-mean-square error of approximation, *CFI* comparative fit index, *IFI* the incremental fit index, *χ*^*2*^*/df ratio* the chi-square/degrees of freedom ratio

### Incremental validity

Characteristics of task, knowledge and social characteristics were positively associated with job satisfaction. Fourteen (out of 21) dimensions of work design were significantly positive associated with job satisfaction, except for physical demands that had a significant negative association. On the other hand, task, knowledge, and social characteristics were negatively associated with job stress. Fifteen (out of 21) dimensions of work design showed a significant negative association with job stress, except for physical demands that presented a significant positive association. Also, job satisfaction and job stress were strongly associated (Table 3).

**Table 3:** Inter-item correlations for items of the Persian Version of WDQ questionnaire

Variable	1	2	3	4	5	6	7	8	9	10	11
1. Work scheduling autonomy	1										
2. Decision-making autonomy	0.66**	1									
3. Work methods autonomy	0.59**	0.61**	1								
4. Task variety	0.49**	0.55**	0.40**	1							
5. Task Significance	0.42**	0.36**	0.47**	0.45**	1						
6. Task identity	0.42**	0.43**	0.45**	0.37**	0.58**	1					
7. Feedback from job	0.31**	0.32**	0.44**	0.29**	0.50**	0.58**	1				
8. Job complexity	− 0.05	0.001	− 0.01	0.22**	0.18**	0.04	0.04	1			
9. Information processing	0.44**	0.48**	0.36**	0.58**	0.52**	0.48**	0.35**	0.28**	1		
10. Problem solving	0.37**	0.45**	0.21**	0.65**	0.36**	0.23**	0.03	0.22	0.60**	1	
11. Skill variety	0.38**	0.48**	0.24**	0.70**	0.34**	0.27**	0.14*	0.32**	0.65**	0.78**	1
12. Specialization	0.33**	0.44**	0.24**	0.54**	0.48**	0.37**	0.25*	0.37**	0.38**	0.66**	0.74**
13. Social support	0.35**	0.27**	0.39**	0.32**	0.42**	0.47**	0.34**	− 0.04	0.38**	0.31**	0.34**
14. Initiated interdependence	0.33**	0.31**	0.35**	0.25**	0.42**	0.58**	0.42**	− 0.01	0.41**	0.21**	0.26**
15. Received interdependence	0.26**	0.24**	0.27**	0.20**	0.38**	0.51**	0.38**	0.02	0.35**	0.18**	0.24**
16. Interaction outside organization	0.42**	0.43**	0.45**	0.58**	0.50**	0.30**	0.22**	0.11	0.41**	0.47**	0.54**
17. Feedback from others	0.41**	0.36**	0.40**	0.27**	0.48**	0.49**	0.38**	0.02	0.37**	0.15**	0.25**
18. Ergonomics	0.31**	0.29**	0.22**	0.37**	0.13*	0.13*	− 0.04	− 0.20**	0.19**	0.39**	0.38**
19. Physical demands	0.10	0.18**	0.16**	0.02	0.13*	0.28**	0.17**	− 0.14*	0.13*	0.11	0.08
20. Work condition	0.33**	0.27**	0.26**	0.30**	0.09	0.06	0.03	− 0.25**	0.06	0.24**	0.23**
21. Equipment use	0.30**	0.42**	0.21**	0.53**	0.26**	0.26**	0.23**	0.12*	0.50**	0.49**	0.60**
22. Job Satisfaction	0.07	0.14*	0.18**	− 0.04	0.18**	0.24**	0.35**	0.004	0.19**	0.21**	− 0.05
23. Perceived stress	− 0.03	− 0.11*	− 0.16*	0.02	− 0.26**	− 0.23**	− 0.31**	− 0.14*	− 0.11*	− 0.20**	− 0.10^*^

Variable	12	13	14	15	16	17	18	19	20	21	22
1. Work scheduling autonomy											
2. Decision-making autonomy											
3. Work methods autonomy											
4. Task variety											
5. Task Significance											
6. Task identity											
7. Feedback from job											
8. Job complexity											
9. Information processing											
10. Problem solving											
11. Skill variety											
12. Specialization	1										
13. Social support	0.38**	1									
14. Initiated interdependence	0.41**	0.47**	1								
15. Received interdependence	0.35**	0.38**	0.92**	1							
16. Interaction outside organization	0.48**	0.27**	0.24**	0.22**	1						
17. Feedback from others	0.38**	0.45**	0.47**	0.42**	0.39**	1					
18. Ergonomics	0.23**	0.30**	0.12	0.06	0.37**	0.25**	1				
19. Physical demands	0.19**	0.10	0.41**	0.37**	0.12*	0.33**	0.06	1			
20. Work condition	0.06	0.26**	0.006	− 0.004	0.35**	0.16**	0.50**	− 0.07	1		
21. Equipment use	0.52**	0.21**	0.35**	0.32**	0.43**	0.35**	0.37**	0.32**	0.22**	1	
22.Job Satisfaction	0.04	0.03	0.27**	0.27**	− 0.01	0.31**	0.22**	− 0.21**	0.15*	0.21**	1
23. Perceived stress	− 0.06	− 0.08	− 0.23**	− 0.22**	− 0.05	− 0.33**	− 0.26**	0.20**	− 0.20**	− 0.18**	− 0.68**

*Correlation is significant at the 0.05 level (2-tailed)

**Correlation is significant at the 0.01 level (2-tailed)

### Convergent/discriminant validity

The results of AVE and MSV are presented in Table 1. Fifteen factors showed adequate levels of divergent validity. The AVE values of 4 out of 21 factors including feedback from job, problem solving, social support, and ergonomics were below 0.50. The MSV values of 3 out of 21 factors including decision-making autonomy, work methods autonomy, and problem solving MSV were above the AVE values. As a set, the questionnaire factors demonstrated good convergent and discriminant validity.

### Reliability

WDQ had an acceptable internal consistency, with a Cronbach’s alpha coefficient ranging from 0.62 to 0.93. Dimensions of ergonomics and skill variety obtained the highest and lowest coefficients, respectively. Corrected item-total correlations were also calculated, which all items presented a significant association with the total score (*P* < 0.001), indicating required consistency. Mean, corrected item-total correlation, Cronbach's alpha, CR, and Cronbach's alpha if item deleted for all items of WDQ are presented in Table 1.

## Discussion

This study evaluated the psychometric characteristics of the Persian version of WDQ using a sample of 270 employers working in different jobs on an industrial estate. A valid, standard method was used for translation and cultural adaptation of the questionnaire. Psychometric properties of the instrument were evaluated using face and content validity, construct validity, concurrent validity, and internal consistency. Face and content validity assess whether or not the instrument measures what it claims and our evaluations used the comments of workers and experts in the fields of ergonomics, occupational health, and industrial and organizational psychologists. Then, where necessary, revisions were made to achieve validity. In addition, quantitative content validity was evaluated using CVI, indicating excellent content validity of all items. Similar results have been reported by studies performed in the USA, Spain, and Brazil [16, 20, 33]. Similar to the original version [16], the Persian version of WDQ could identify expected differences in job characteristics in various job categories.

Consistent with previous studies in the USA, Spain, and Brazil [16, 20, 33], the CFA results indicated that the 21-factor model had the highest fit. While 18-, 19-, and 20-factor models were also confirmed, the 21-factor model presented the best-fit indicators, which is consistent with previous studies in other countries [16, 20, 33, 34]. Regarding the profound changes in psychological-social indicators of Iranian workers and observed changes in industries and job positions of the organizations, these results were not unexpected. In the past 4 decades (since the 1979 revolution), Iranian workers’ education and skill levels have considerably increased, leading to changes at managerial levels, particularly social characteristics and knowledge of managers.

Desirable reliability of the Persian version of the WDQ was confirmed by the Cronbach’s alpha (0.87), which was consistent with the coefficient reported for the original version (0.87) [16], and similar to other studies performed in Spain [20] and Brazil [33]. Of all 21 job characteristics, only ergonomics and feedback from the job obtained a coefficient lower than 0.7. Although a Cronbach’s alpha from 0.6 to 0.7 is the acceptable level of reliability [35, 36]. Regarding internal consistency, the item-total correlation was used for all 21 job characteristics. With the exception of items 24 (from *Feedback from Job*) and 66 (from *Ergonomics*), the item-total correlation all the items of WDQ were acceptable. Similarly, the Cronbach's alpha has been not appropriate for the *Ergonomics* dimension in other studies [16, 37, 38]. Some insight into this can be gained from considering the three items that make up the *Ergonomics* dimension. Two items are positively worded and one is negative (i.e. “The job involves excessive reaching”). The item in particular, can be effective in reducing the Cronbach's alpha of the *Ergonomic*s dimension. It asks about the level of reaching, and its meaning has the potential to vary from one person to another, leading to ambiguities.

This study demonstrated a significant association between the scales of WDQ with job satisfaction and job stress, indicating appropriate concurrent validity of the Persian version of WDQ. Bayona et al. [38] also reported similar results. According to Hackman and Oldham’s [13] job characteristics theory, emphasizing job characteristics can increase job satisfaction and decrease stress among staff [13]. In this study, stress and job satisfaction variables showed a negative and positive association with WDQ dimensions, respectively.

### Limitations

It is necessary to mention some limitations of our study. Self-report instruments are prone to bias, even we removed items related to department and job title to minimize the bias. Another limitation was the cross-sectional design of the study, with its limitations in investigating cause-and-effect relationships. In addition, this study was conducted only in one city, and because of the culture of Iranian society, women’s employment is limited, and few women participated in this study. The consequence of this is not great, as the purpose of the study was to develop a tool for use in Iranian society.

## Conclusion

This study demonstrated the appropriate psychometric characteristics of the Persian version of WDQ. Hence, WDQ is a valid and reliable instrument to assess work characteristics among Iranian employees. Similar to other studies which have looked at the psychometric characteristics of the WDQ, the 21-factor version showed better-fit indicators. Accordingly, the authors recommend its administration in future studies in Iran. The work characteristics were significantly associated with job satisfaction and job stress. Therefore, improved work design would reduce negative consequences, such as job stress, and increase positive behaviors, such as job satisfaction.

## Data Availability

The datasets used and/or analysed during the current study are available from the corresponding author on reasonable request.

## References

[1] Grant AM, Parker SK (2009). 7 Redesigning work design theories: the rise of relational and proactive perspectives. Acad Manag Ann.

[2] Richard HJ, Oldham G (1976). Motivation through the design of work: test of a theory. Organ Behav Hum Perform.

[3] Güntert ST (2015). The impact of work design, autonomy support, and strategy on employee outcomes: a differentiated perspective on self-determination at work. Motiv Emot.

[4] Boatright CM (2014). A quantitative examination of the effect of work design on turnover intention of information technology professionals.

[5] Steyn R, Vawda N (2014). 7 Redesigning work design theories: the rise of relational and proactive perspectives. Job characteristics: their relationship to job satisfaction, stress and depression. J Psychol Afr.

[6] Ganster DC, Fox ML, Dwyer DJ (2001). Explaining employees' health care costs: a prospective examination of stressful job demands, personal control, and physiological reactivity. J Appl Psychol.

[7] Smith A. An inquiry into the nature and causes of the wealth of nations. London: W. Strahan and T Cadell; 1776.

[8] Babbage C. On the economy of machinery and manufactures. London: Knight; 1835.

[9] Morgeson FP, Humphrey SE (2008). Job and team design: toward a more integrative conceptualization of work design. Res Pers Hum Resour Manag.

[10] Ewen RB, Smith PC, Hulin CL (1966). An empirical test of the Herzberg two-factor theory. J Appl Psychol.

[11] Morgeson FP, Delaney-Klinger K, Hemingway MA (2005). The importance of job autonomy, cognitive ability, and job-related skill for predicting role breadth and job performance. J Appl Psychol.

[12] Parker SK, Wall TD, Cordery JL (2001). Future work design research and practice: towards an elaborated model of work design. J Occup Organ Psychol.

[13] Hackman JR, Oldham GR (1975). Development of the job diagnostic survey. J Appl Psychol.

[14] Campion MA (1988). Interdisciplinary approaches to job design: a constructive replication with extensions. J Appl Psychol.

[15] Edwards JR, Scully JA, Brtek MD (2000). The nature and outcomes of work: a replication and extension of interdisciplinary work-design research. J Appl Psychol.

[16] Morgeson FP, Humphrey SE (2006). The Work Design Questionnaire (WDQ): developing and validating a comprehensive measure for assessing job design and the nature of work. J Appl Psychol.

[17] Stegmann S, van Dick R, Ullrich J, Charalambous J, Menzel B, Egold N, et al. Der work design questionnaire. Zeitschrift für Arbeits-und Organisationspsychologie A&O. 2010.

[18] Chiou H, Chou J, Lin P. Validation of the Work Design Questionnaire and latent class analysis of work structure. 測驗學刊. 2010;57:139–79.

[19] Hauk M. Kwestionariusz Cech Pracy–opracowanie polskiej wersji narzędzia do badania cech pracy i środowiska zawodowego. Wstępne wyniki badań. Acta Univ Lodz Folia Psychol 2014(18):129–45.

[20] Ríos MF, Vielma RGR, García JCS, Aravena MB, Vargas JDP, Díaz MÁR. Spanish-language adaptation of Morgeson and Humphrey’s Work Design Questionnaire (WDQ). Span J Psychol. 2017;20. 10.1017/sjp.2017.24 28595664

[21] Beaton DE, Bombardier C, Guillemin F, Ferraz MB (2000). Guidelines for the process of cross-cultural adaptation of self-report measures. Spine.

[22] Polit DF, Beck CT, Owen SV (2007). Is the CVI an acceptable indicator of content validity? Appraisal and recommendations. Res Nurs Health.

[23] DeVellis RF (2016). Scale development: theory and applications.

[24] Rajabi F, Mokarami H, Cousins R, Jahangiri M (2022). Structural equation modeling of safety performance based on personality traits, job and organizational-related factors. Int J Occup Saf Ergon.

[25] Maroufizadeh S, Zareiyan A, Sigari N (2014). Reliability and validity of Persian version of perceived stress scale (PSS-10) in adults with asthma. Arch Iran Med.

[26] Wongpakaran N, Wongpakaran T (2010). The Thai version of the PSS-10: an investigation of its psychometric properties. BioPsychoSoc Med.

[27] Baik SH, Fox RS, Mills SD, Roesch SC, Sadler GR, Klonoff EA, Malcarne VL (2019). Reliability and validity of the Perceived Stress Scale-10 in Hispanic Americans with English or Spanish language preference. J Health Psychol.

[28] Bowling NA, Hammond GD (2008). A meta-analytic examination of the construct validity of the Michigan Organizational Assessment Questionnaire Job Satisfaction Subscale. J Vocat Behav.

[29] Hair JF, Anderson RE, Babin BJ, Black WC (2010). Multivariate data analysis: A global perspective.

[30] Michielsen HJ, De Vries J, Van Heck GL, Van de Vijver FJ, Sijtsma K (2004). Examination of the dimensionality of fatigue. Eur J Psychol Assess.

[31] Abdi F, Jahangiri M, Kamalinia M, Cousins R, Mokarami H (2021). Presenteeism and work ability: development of the Persian version of the Stanford Presenteeism Scale (P-SPS-6) and measurement of its psychometric properties. BMC Psychol.

[32] Byrne BM (2016). Structural equation modeling with AMOS: basic concepts, applications, and programming.

[33] Borges-Andrade JE, Peixoto ALA, Queiroga F, Pérez-Nebra AR (2019). Adaptation of the work design questionnaire to Brazil. Rev Psicol Org Trabalho.

[34] Stegmann S, Dick RV, Ullrich J, Charalambous J, Menzel B, Egold N (2010). Der work design questionnaire. Z Arbeits Organisationspsychol.

[35] Taber KS (2018). The use of Cronbach’s alpha when developing and reporting research instruments in science education. Res Sci Educ.

[36] Nazari M, Beigi R, Salesi M, Cousins R, Mokarami H (2020). Development and validation of the tool for the evaluation of the behavioral factors affecting the prevalence of musculoskeletal disorders in Iranian students. BMC Pediatr.

[37] Fernández Ríos M, Ramírez Vielma RG, Sánchez García JC, Bargsted Aravena M, Polo Vargas JD, Ruiz Díaz MÁ (2017). Spanish-Language adaptation of Morgeson and Humphrey’s Work Design Questionnaire (WDQ). Span J Psychol.

[38] Bayona JA, Caballer A, Peiró J-M (2015). The Work Design Questionnaire: Spanish version and validation. Rev Psicol Trabajo Org.

